# Novel Protocol for the Preparation of Porcine Bone Marrow Primary Cell Culture for African Swine Fever Virus Isolation

**DOI:** 10.3390/mps6050073

**Published:** 2023-08-24

**Authors:** Olga Puzankova, Vera Gavrilova, Roman Chernyshev, Ivan Kolbin, Alexey Igolkin, Alexandr Sprygin, Ilya Chvala, Ali Mazloum

**Affiliations:** FGBI Federal Centre for Animal Health, 600901 Vladimir, Russia; puzankova@arriah.ru (O.P.); gavrilova@arriah.ru (V.G.); chernishev_rs@arriah.ru (R.C.); kolbin@arriah.ru (I.K.); igolkin_as@arriah.ru (A.I.); sprygin@arriah.ru (A.S.); chvala@arriah.ru (I.C.)

**Keywords:** porcine bone marrow, primary cell culture, African swine fever, virus isolation

## Abstract

Isolation of African swine fever virus (ASFV) is a critical step towards the identification, titration, characterization, and even modification of the virus. Therefore, it is important to identify a suitable cell line that supports the efficient replication of ASFV for these purposes. This should be achieved even when starting with a low virus load, as in the case of isolating the virus from field samples. This article presents a detailed protocol on the preparation of porcine bone marrow primary (PBMP) cell culture, which has a high sensitivity towards ASFV, resulting in high viral yields with a minimal risk of bacterial contamination.

## 1. Introduction

African swine fever virus (ASFV) is the only member of the *Asfarviridae* family genus *Asfivirus* [1]. It is the causative agent of ASF disease, a highly contagious hemorrhagic fever of domestic pigs and wild boars, which has a mortality rate of up to 100% [2]. The virus has a double-stranded DNA genome around 170–197 kilo-base pair (kbp) in length, encoding roughly 160 ORF [3]. Laboratory confirmation of the presence of the virus in a sample (usually whole blood, spleen, or bone marrow) is based on a real-time polymerase chain reaction (RT-PCR) in order to detect selected regions of the viral genome, followed by virus isolation (VI) in cell culture, according to the World Organization for Animal Health (WOAH) recommendations [4].

Due to the observed phenomenon of “hemadsorption” represented by the accumulation of red blood cells (RBCs) at the surface of infected cells, the first biological models for ASF virus isolation were cell cultures of the mononuclear-macrophage series (blood leukocytes and bone marrow macrophages of pigs) [5,6,7,8]. Porcine kidney (PK) continuous cell lines (PK2a, PK-15, PK-13, and IB-RS-2) and primary cultures required additional ASF virus adaptation by serial passaging (from 8 to 73 passages) to achieve a high level of viral load and the appearance of a cytopathic effect (CPE) [7,8,9,10,11]. Monolayer cultures of porcine kidney cells as well as chicken embryo fibroblasts and BHK-21 were used to study the morphology and the replication of the ASF virus by plaque and electron microscopy methods, but they were not suitable for the primary isolation and identification of field isolates [12,13,14,15,16]. Adaptation of the virulent ASF virus of genotype I (for example, Lisbon 60, BA71, and others) to green monkey kidney cell lines Vero and CV-1 led to the appearance of attenuated strains used in the production of antigens and sera for the manufacturing of diagnostic biological products [17,18,19,20,21]. A comparative analysis of the sensitivity of 16 different cell cultures to the ASF virus was performed, before showing the ability of ASFV to replicate in cells derived from *Sus scrofa* (alveolar macrophages), green monkey (Vero, CV2, COS-1), and rhesus monkey (LLCMK2) [22]. While in cell cultures derived from hamster (BHK-21, CHO), human (HeLa, Sh-SY5Y), mouse (A 9, 3T6, NP3, L 929), dog (MDCK), and fall armyworm (Sf9), the virus did not replicate [22]. Hurtado et al. showed the sensitivity of COS-1 cells obtained by immortalizing CV-1 with the SV40 virus to both virulent strains of the ASF virus (E70, Malawi 82, Lisbon 60, and others) and attenuated ones (ΔEP153R, NHV, BA71V, and others) [23]. Masujin et al. obtained a continuous cell culture of porcine renal macrophages (IPKM) sensitive to virulent strains of the ASF virus (Armenia07, Kenya05/Tk-1, and Espana75) [24].

The majority of laboratories and research institutes investigating and performing routine diagnostics on ASFV employ three main cell culture lines. These are primary macrophage cultures prepared from fresh pig blood (PBMC), primary alveolar macrophage cultures (PAMC), and continuous cell culture MA-104 [4,25,26,27]. PBMC and PAMC are the most sensitive to the virus infection and replication since they are derived from the natural host, but they are time-consuming to prepare and they often result in a low cell yield, and researchers also frequently report fungal contamination when preparing PAMC. The MA-104 cell culture has been tested using several ASFV isolates, but they are not suitable for laboratory diagnosis when the virus load is low and requires sensitive original host cells. Additionally, the successful isolation of viruses belonging to new genotypes containing new genomic mutations or even novel recombinants (for example, the novel genotype I and II recombinants recently described in China) has not been tested in continuous cell lines [28]. 

In this paper, we describe a step-by-step procedure for the preparation of porcine bone marrow primary (PBMP) cell culture that has a high yield and high sensitivity as well as a low risk of contamination. 

## 2. Equipment

1. Class II biological safety cabinet (model: Streamline—E Series, catalog number: SC2-E).
2. Humidified 37 ± 2 °C, 5 ± 1% CO_2_ incubator (model: S-Bt Smart Bio Term, catalog number: BS-010425-A01).
−70 °C freezer (Sanyo MDF-U53V, catalog number: 9782).
−20 °C freezer (model: LG, catalog number: GA-B419SQQL).
5. Refrigerator, 4 °C, with an acceptable range of 2 °C to 8 °C (model: LG, catalog number: GA-B419SQQL).
6. Water bath, 37 °C (model: WB-4MS Stirred water bath, BioSan, catalog number: BS-010406-AAA).
7. Portable Pipettor (Dragon Laboratory Instruments Limited, Beijing, China, Levo plus, catalog number: 710932).
8. Micropipettors: Single channel, 1–10 µL, 2–20 µL, 50–200 µL, 100–1000 µL (Eppendorf catalog numbers: Q11390H, K12770H, Q24196H, L18745H).
9. Micropipettors: Multi-channel, 0.5–10 µL, 5–50 µL, 50–300 µL (BTLab systems, Saint Louis, USA, Lab System, catalog numbers: N87539, D23745, E21512).
10. Multi-channel for larger volumes (Rainin^TM^ Pipet-Life XLS, L-1200, 100–1200 µL, catalog number: 17014497).
11. Inverted Microscope (Euromex Microscopen, Arnhem, Netherlands, model: Olympus Euromex Oxion, catalog number: SKU: EOX.2053-PLPH).
12. Tabletop Centrifuge (HERMLE Labortechnik GmbH, Wehingen, Germany, model: HERMLE Z 306, catalog number: 310.00 v01).
13. Shaker incubator (BioSan, Riga, Latvia, model: Biosan ES-20, catalog number: BS-010135-AAA).
14. Electronic balance (model: OHAUS Navigator NV212, catalog number: 83033081).
15. Countess™ automatic cell counter.

## 3. Materials and Reagents

1. Pipette tips: 10 μL, 20 μL, 200 μL, 1000 μL (Thermo Fisher Scientific, Waltham, MA, USA, catalog numbers: 2140-05, 72830-440, 72830-044, 72830-042).
2. Sterile serological pipettes: 1 mL, 5 mL, 10 mL, 25 mL, 50 mL (Thermo Fisher Scientific, catalog numbers: 14672-918, 14672-920, 4101, 14672-900, 53384-451).
3. Conical tubes: 15 and 50 mL (Corning, AZ, USA, Falcon^®^, catalog numbers: 353110 and 352098)
4. T-75 flask (Corning, catalog number: 430641U).
5. T-25 flask (Corning, catalog number: 430825).
6. T-225 flask (Corning, catalog number: 431082).
7. Millex-HV Syringe Filter Unit, 0.45 µm, PVDF, 33 mm, gamma sterilized (Catalog number: SLHV033RS).
8. Absorbent paper (Kimtech Pure CL4, Kimberly-Clark Professional, GA, USA, catalog number: 7646).
9. Reservoirs (Costar Reagent Reservoir, Corning^®^, catalog number: 29442-474).
10. TrypLE™ Express Enzyme (1X), no phenol red (Thermofisher, catalog number: 12604-013).
11. EZFlow^®^ cell strainers (catalog number 410-0002-OEM).
12. Minimum Essential Medium (MEM) (Thermofisher, catalog number: 11095-080).
13. ASFV field strain: For example an ASFV-Arm07 field strain (Arm07).
14. Trypan blue solution, 0.4% (Thermofisher scientific, catalog number: 15250061).
15. Gentamicin 40mg/ml Solution (Wockhardt UK Ltd. Wrexham, UK, catalog number: J01GB03).
16. Penicillin G sodium salt (Sigma 1000000 Units, Sigma-Aldrich, Sofia, Bulgaria, Catalog Number P3032-1MU).
17. Streptomycin sulfate (Sigma, Cat. Number 5711-100GM).
18. Saline solution (Sigma-Aldrich, catalog number: S8776).
19. Bacto™ TC Lactalbumin Hydrolysate (Thermofisher, catalog number 259962).
20. 70% Ethanol.
21. Fetal Bovine Serum (Sigma-Aldrich, Cat. Number F7524-500ML).

## 4. Animals

Clinically healthy piglets, 4–6 weeks of age (8–15 kg), obtained from farms free from ASF, CSF, and other infectious diseases, are used. Animals were housed for one week before necropsy and monitored twice a day by veterinary staff. Water and feed were provided ad libitum.

Necropsy must be performed in accordance with BSL-3 guidelines and the approved statement from the Animal Ethics Committee.

## 5. Procedure

### 5.1. Selection of Tubular Bones

For the preparation of the cell culture, large tubular bones (humerus, radius, ulna, fibula, femur bone, and tibia) are taken during necropsy.

Sterile containers with samples are placed in a sterile waterproof metal lab container and transferred to the laboratory, where all subsequent stages of the cell culture preparation are performed.

### 5.2. Isolation of PBMP Cells

**Figure 1:**
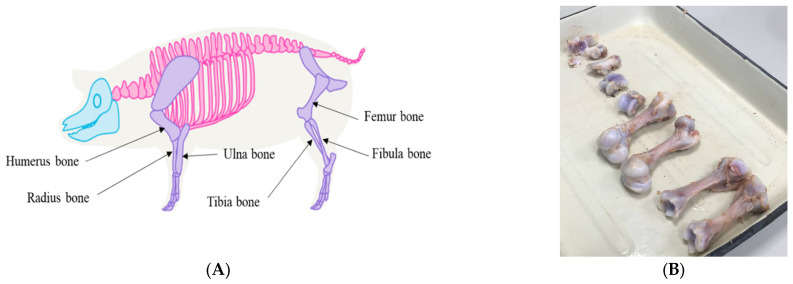
Tubular bones used for the preparation of PBMP cell culture. (**A**) Skeletal scheme of domestic pig. (**B**) Tubular bones after removal of muscle and tissue mass.

**Figure 2:**
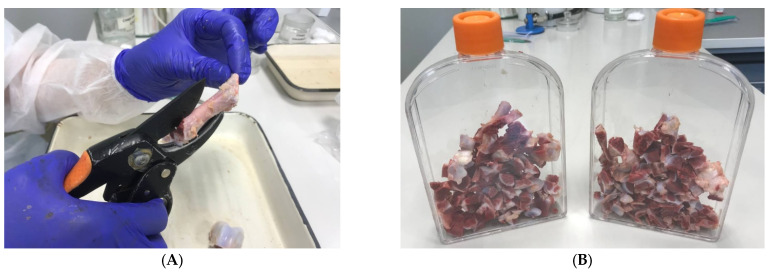
Processing of tubular bones before washing. (**A**) Process of cutting bone into pieces for bone-marrowexposure. (**B**) Bone pieces in T-225 plastic culture flask.

1. Place isolated tubular bones (humerus, ulna, radius, femur, and tibia) in a vessel and wash with 70% ethanol by incubating in a shaker-incubator at room-temperature and 250 rpm for 10–15 min in order to disinfect the surfaces of the samples (Figure 1A).
2. Remove the muscle and tissue mass from the bones with a surgical scalpel and rewash the bones, similar to step 1 (Figure 1B).

3. Cut the bones with a sterile hand pruner into small cubes approximately 2 cm^2^, thereby exposing the bone marrow as much as possible (Figure 2A).
4. Place the bone pieces uniformly in a T-225 plastic culture flask (Figure 2B).

5. Add washing buffer (Table 1) to the bone pieces in a ratio of 1:1 (*m*/*v*), after which the vials are carefully inverted 1–2 times. Incubate at room temperature (RT) for 2 min, and then discard the buffer.

6. Add saline solution in a ratio of 1:1.5 (*m*/*v*) into the flasks with bone pieces, and incubate in a shaker-incubator at 37 °C and 250 rpm for 20 min to remove the bone marrow cells from the bone cavities.
7. Filter the cell suspension through an 8-layer medical gauze filter into a sterile glass vial (about 120 mL each). Step 6 can be repeated in order to gather more cells from the bone marrow. The filtrate is evenly poured into sterile centrifuge beakers and centrifuged at +4 °C and 1400 rpm for 20 min.
8. After centrifugation, discard the supernatant and re-suspend the pellet in growth medium (Table 1) (about 25 mL for one glass vial). The cell suspension is successively passed through an 8-layer medical gauze filter and through nylon cell strainers with a mesh size of 100 and 70 µm.
9. Calculate the cell concentration using a Countess^TM^ automatic cell counter or analogue (ex. cell suspension sample is diluted 10 times in saline solution, and 0.4% trypan blue solution is added to the diluted volume in a ratio of 1:1 and loaded into the chamber of a disposable counter slide.)

Cell concentration is calculated by the formula:

X = H × 2 × 10;

where X is the number of cells in 1 cm^3^ of cell suspension; H is the number of living cells indicated by the device in 1 cm^3^ of a cell suspension sample; 2 is the coefficient of cell suspension dilution with 0.4% trypan blue solution; and 10 is the coefficient of dilution of the cell suspension with the saline solution

10. Dilute the cell suspension in growth medium, and adjust to an inoculation concentration of 20–30 million cells/cm^3^.
11. Add the diluted suspension of PBMP cells to cell culture flasks as indicated in Table 2.

12. Incubate the flasks in a humidifier at +37 °C with 5% CO_2_ for 48 h.
13. After a 48-h incubation, the growth medium is replaced with maintenance medium (Table 1) as shown in Table 3.

**Figure 3:**
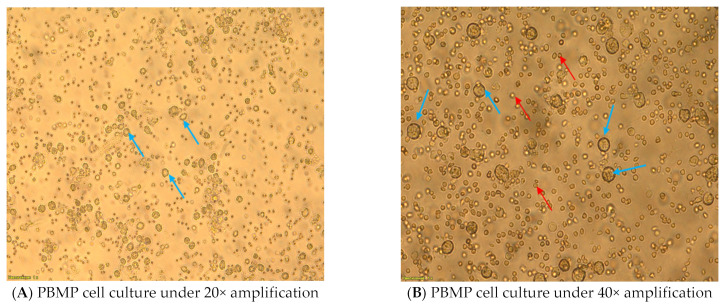
Porcine bone marrow primary cell culture 48 h after preparation in T-75 flask. Blue arrow: porcine bone marrow macrophages. Red arrow: porcine red blood cells.

After changing the medium, cell growth is assessed under the microscope. PBMP macrophages should be round or oval and 40–60 microns in size with a smooth surface and a high density of attachment to the substrate, and they should be located singly and not form coagulates (Figure 3). A cell culture that does not adhere to the previously mentioned characteristics is discarded. 

PBMP cell culture should be used, depending on the purpose of the study, within 2–5 days after adding maintenance medium.

## 6. Expected Results (Isolation of African Swine Fever Virus from Biological Samples)

Cell culture prepared in the laboratory can be in cell culture plates (48-wells or 96-wells) or in cell culture flasks such as T-25 or T-75, depending on the needs of the researchers. Plates are used for virus titration, while flasks can be used for the isolation of new viruses from biological materials or the accumulation of viruses for further uses, such as full genome sequencing. 

### 6.1. Sample Preparation and Infection

Organ samples (spleen, whole blood, lungs, lymph nodes, or kidney) taken from infected animals or corps (wild boars or domestic pigs suspected of ASF) are homogenized, and a suspension is prepared by adding saline solution in a ratio of 1:10 (*m*/*v*), followed by centrifugation at 1100 g for 10 min. The supernatant is filtered through a Millex-HV Syringe filter (0.45 µm). Gentamicin sulfate (40mg/mL) is added to the filtrate at a final concentration of 0.01% and incubated for 1 h at a temperature ±37 °C, followed by inoculation into porcine PBMP cell culture. The flasks can be incubated up to 7 days at 37 °C in a 5% humidified CO_2_ incubator. The results are estimated under the microscope on a daily basis for the presence of hemadsorption. 

### 6.2. ASF Virus Identification

The cell culture is examined for the presence of hamadsorption under a low magnification microscope (10 × 10). The first observation is performed 15–18 h after inoculation, and, subsequently, on a daily basis for 7 days. Before examination, the cell culture should be shaken slightly so that erythrocytes that are not attached to the surface of the cells will be evenly distributed in the culture medium.

**Figure 4:**
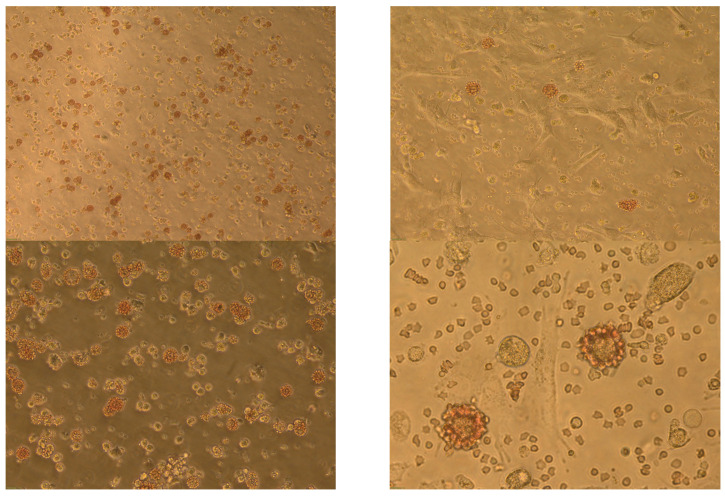
Hemadsorption in PBMP cell culture infected with log10^3^ HAU of ASFV isolate Arm07 48 h after infection (different amplifications 10×, 20×, 40×, and 60×).

The hemadsorption phenomenon is based on the attachment of erythrocytes to the surface of virus-infected macrophages. The result for ASF is considered positive in the presence of haemadsorption (Figure 4).

If there are no manifestations of hemadsorption in the inoculated cell culture during the observation period, it is necessary to conduct two additional passages in the PBMP cell culture. After the third passage, if no hemadsorption is observed, the VI result is considered negative (absence of the virus, or inactivated virus). Taking into consideration that not all ASFV are hemadsorbing, if no hemadsorption is detected, VI must be accompanied by RT-PCR in order to detect the virus and confirm its replication by Ct level.

## 7. Validation

To validate this cell culture for ASFV isolation and titration, different viruses were used to infect PBMP and porcine spleen cell culture (PSC) (n = 3) [29,30]. The viruses used included Arm07 (Genotype II), ASFV/ARRIAH/CV-30 (Genotype II, adapted to CV-1 cell culture by performed 30 consecutive passages) [29], Lisbon 60 (Genotype I), K49 (Genotype I), and Mozambique-78 (Genotype V), as well as 18 other isolates taken from different regions of the Russian Federation and all representing Genotype II (Supplementary Table S1). The 96-well cell culture plates were infected at 100 HADU_50_/cm^3^, and titer (in lg HADU_50_/cm^3^) was calculated on the fifth day after inoculation as described by de Leon et al. in 2013 [4]. The results are represented in Supplementary Table S1. 

## 8. Conclusions

African swine fever virus isolation in cell culture is a critical step in virus characterization and the study of its biological properties. The rate of mutation in the virus genome due to its natural circulation between susceptible animals resulted in the appearance of 24 genotypes and a novel recombinant variant [31,32,33]. It is therefore essential to select the appropriate cell culture with high sensitivity and yield for VI. In recent studies, alveolar and blood origin macrophage cell culture preparation protocols have been described [4,5,6,7,8,9]. Moreover, continuous MA-104 cell culture has been recommended for VI, but its sensitivity on field isolates has not been tested. In this paper, we have described the protocol for preparing a PBMP cell culture with a high yield and sensitivity for studying and isolating any virus isolate. 

## Figures and Tables

**Table 1:** Reagents prepared right before starting the protocol.

Wash Buffer (1×)		
	Volume Added	Final Concentration
Saline solution	Up to 500 mL	
Gentamicin 40 mg/mL Solution	10 mL	1.6 mg/mL (1.6%)
Streptomycin sulfate	1g	0.1%
Penicillin G sodium salt	10^6^ Units	10^3^ Units
**Growth Medium**		
	**Volume Added**	**Final Concentration**
Minimum Essential Medium (MEM)	Up to 1000 mL	
Lactalbumin hydrolysate	2.5 g	0.25%
Fetal Bovine Serum (FBS)	150 mL	15%
Gentamicin 40 mg/mL Solution	2 mL	0.08 mg/mL (0.08%)
**Maintenance Medium**		
	**Volume Added**	**Final Concentration**
Minimum Essential Medium (MEM)	Up to 1000 mL	
Lactalbumin hydrolysate	2.5 g	0.25%
Fetal Bovine Serum (FBS)	50 mL	5%
Gentamicin 40 mg/mL Solution	2 mL	0.08 mg/mL (0.08%)

**Table 2:** Amount of PBMP cell culture (cell/cm^3^) to be added to cell culture flasks (area in cm^2^).

	Area (cm^2^)	Cell/cm^3^
Flask		
T-25	25	10–15 × 10^7^
T-75	75	30–45 × 10^7^
T-225	225	15–20 × 10^8^
Culture plates		
48-well	1.1	6–10 × 10^7^
96-well	0.32	2–3 × 10^7^

**Table 3:** Minimum volume of maintenance medium (mL) to be added to the cell-culture flask (area in cm^2^).

	Area (cm^2^)	Volume (mL)
Flask		
T-25	25	3–5
T-75	75	8–15
T-225	225	45–68
Culture plates		
48-well	1.1	0.2–0.4
96-well	0.32	0.1–0.2

## Supplementary Materials

The following supporting information can be downloaded at: https://www.mdpi.com/article/10.3390/mps6050073/s1, Table S1: Results of validating PBMP cell culture for ASFV isolation and titration (n = 3).
