# Supplementary material for: Novel Protocol for the Preparation of Porcine Bone Marrow Primary Cell Culture for African Swine Fever Virus Isolation
Source: Methods Protoc. 2023 Aug 24;6(5):73. doi: 10.3390/mps6050073 (PMC10514816; doi:10.3390/mps6050073)
Supplement: Supplementary file 1 [file mps-06-00073-s001.zip › mps-2480350-supplementary.pdf]

Table S1. Results of validating PBMP cell culture for ASFV isolation and titration (n=3).

No.	Virus Isolate	Region of isolation	Genotype	Titer (lg HADU50/cm3 ±SD)	
				PSC cell culture	PBMP cell culture
1	Arm 07	Armenia	Genotype II	6.80±0.10	7.10±0.30
2	ASFV/ARRIAH/CV-30	Adapted to CV-1 cell culture	Genotype II	6.93±0.15	6.63±0.15
3	Lisbon 57	Portugal	Genotype I	6.60±0.23	7.20±0.20
4	K49	Congo	Genotype I	7.75±0.25	7.90±0.10
5	Mozambique-78	Mozambique	Genotype V	6.50±0.40	6.90±0.15
6	Amur/2022	Amur	Genotype II	6.8±0.30	7.0±0.47
7	ASFV/Primorsky 19/WB-6723	Primoriya	Genotype II	7.02 ± 0.12	7.35±0.25
8	ASFV/Amur 19/WB-6905	Amur	Genotype II	6.80 ± 0.45	6.55±0.10
9	ASFV/Ulyanovsk 19/WB-5699	Ulyanovsk	Genotype II	7.21 ± 0.41	6.90±0.40
10	ASFV/Kabardino-Balkaria 19/WB-964	Kabardino-Balkaria	Genotype II	7.40 ± 0.14	7.50±0.20
11	Astrakhan/2022	Astrakhan	Genotype II	6.85±0.20	7.30±0.07
12	Vladimir/2022	Vladimir	Genotype II	6.55±0.15	7.25±0.17
13	Kaliningrad/2022	Kaliningrad	Genotype II	7.10±0.33	6.90±0.27
14	Omsk/2022	Omsk	Genotype II	6.50±0.10	6.25±0.13
15	Penza/2022	Penza	Genotype II	6.22±0.15	6.75±0.17
16	Tatarstan/2022	Tatarstan	Genotype II	6.55±0.31	6.40±0.33
17	Tver/2022	Tver	Genotype II	7.20±0.10	7.00±0.07
18	Ulyanovsk/2022	Ulyanovsk	Genotype II	5.90±0.44	6.10±0.07
19	Samara/2022	Samara	Genotype II	6.40±0.20	6.75±0.13
20	Saratov/2022	Saratov	Genotype II	6.80±0.25	7.10±0.24
21	Orel/2022	Orel	Genotype II	7.10±0.10	7.40±0.27
22	Kursk/2022	Kursk	Genotype II	7.60±0.20	8.05±0.17
